# miRNAs in Glomerular Diseases: From Pathogenic Insight to Therapeutic Potential: A Narrative Review

**DOI:** 10.3390/cells15020094

**Published:** 2026-01-06

**Authors:** Mugurel Apetrii, Alexandru Dan Costache, Irina Iuliana Costache Enache, Luminita Voroneanu, Andreea Simona Covic, Mehmet Kanbay, Dragos Viorel Scripcariu, Adrian Covic

**Affiliations:** 1 “Grigore T. Popa” University of Medicine and Pharmacy, 700115 Iasi, Romania; mugurel.apetrii@gmail.com (M.A.); ii.costache@yahoo.com (I.I.C.E.); lumivoro@yahoo.com (L.V.); andreea.covic@gmail.com (A.S.C.); dscripcariu@gmail.com (D.V.S.); accovic@gmail.com (A.C.); 2 “Dr. C.I. Parhon” University Hospital, 700503 Iasi, Romania; 3 Clinical Rehabilitation Hospital, 700661 Iasi, Romania; 4 “St. Spiridon” Emergency County Hospital, 700111 Iasi, Romania; 5 School of Medicine, Koç University, 34450 Istanbul, Turkey; drkanbay@yahoo.com; 6 Regional Institute of Oncology, 700483 Iasi, Romania

**Keywords:** microRNA, glomerulopathies, lupus nephritis, diabetic nephropathy

## Abstract

This article explores the multifaceted role of micro-ribonucleic acids (RNAs) (miRNAs) as critical posttranscriptional regulators in renal physiology and disease, with a focus on their emerging significance in glomerulopathies. miRNAs, small endogenous noncoding RNAs, modulate gene expression by promoting messenger RNA degradation or inhibiting translation, thereby orchestrating essential cellular processes such as proliferation, differentiation, apoptosis, and stress responses. Recent advances have revealed that aberrant miRNA expression profiles are intricately linked to the pathogenesis and progression of various renal diseases, including acute kidney injury, chronic kidney disease, alloimmune injury in solid organ transplantation and glomerulonephritis. This review summarizes the pathogenic and protective roles of miRNAs in major glomerulopathies, discusses their potential as diagnostic and prognostic biomarkers, and outlines future directions for their integration into personalized therapeutic strategies. At the moment, it is not fully established whether some of these mechanisms are the primary pathogenic driver or a secondary response. Combining miRNAs with other molecular markers may further enhance diagnostic and predictive accuracy, facilitating clinical translation, while selective targeting of specific miRNAs at different stages of disease progression could offer promising therapeutic opportunities.

## 1. Introduction

Micro ribonucleic acids (RNAs) (miRNAs) are endogenous, small noncoding RNAs (~21–25 nucleotides) that play key roles in RNA interference (RNAi), a conserved mechanism present in all organisms that regulates gene transcription and post-transcriptional messenger RNA (mRNA) processing. Rather than encoding proteins, miRNAs bind target mRNAs to repress translation or promote mRNA degradation, thereby shaping cellular phenotypes. First discovered in Caenorhabditis elegans in the early 1990s, miRNAs are now recognized as central components of gene regulatory networks involved in a wide range of physiological and pathological processes. A single miRNA can regulate multiple genes, and multiple miRNAs can converge on the same gene, adding significant regulatory complexity. Because of their ability to selectively modulate gene expression, miRNAs and other RNAi-based approaches are powerful tools in basic research and promising candidates for targeted therapeutics [1].

miRNAs orchestrate a wide array of biological processes by fine-tuning gene expression networks critical for cellular homeostasis. They regulate cell cycle progression, differentiation, apoptosis, and stress responses. During embryonic development, specific miRNAs contribute to lineage specification by modulating transcription factor networks that govern stem cell pluripotency and differentiation into specialized cell types, including myocytes, neurons, and immune cells. The temporal and spatial regulation of gene expression by miRNAs is essential for normal organismal development and tissue homeostasis [2].

Aberrant miRNA expression profiles have been linked to various pathologies. Oncogenic miRNAs can promote tumorigenesis by downregulating tumor suppressor genes, whereas tumor-suppressive miRNAs inhibit oncogenes. Dysregulated miRNA expression contributes to cancer initiation, progression, metastasis, and chemoresistance [3,4].

Beyond oncology, altered miRNA patterns are associated with autoimmune diseases, cardiovascular pathologies such as atherosclerosis and heart failure, and neurodegenerative disorders, including Alzheimer’s and Parkinson’s disease. The tissue-specific expression and stability of miRNAs in bodily fluids also underscore their potential as diagnostic biomarkers and therapeutic targets [3,4].

In nephrology, miRNAs have gained considerable attention for their roles in kidney development, homeostasis, and disease. The kidney’s complex cellular architecture and dynamic response to environmental stimuli are tightly regulated by miRNA-mediated gene expression control. miRNAs modulate key processes such as cellular differentiation, proliferation, apoptosis, and fibrosis within renal tissues [2,5].

Furthermore, aberrant miRNA expression profiles have been implicated in the pathogenesis of a spectrum of kidney disorders, including acute kidney injury (AKI), chronic kidney disease (CKD), diabetic nephropathy, and glomerulonephritis [2,5].

The mechanistic insights into miRNA biogenesis and function have unveiled their contribution to renal pathophysiology at multiple levels, from modulating inflammatory pathways and extracellular matrix remodeling to influencing epithelial-to-mesenchymal transition (EMT). Moreover, circulating and urinary miRNAs have emerged as promising minimally invasive biomarkers for early detection, prognosis, and therapeutic monitoring of renal diseases [6].

**Figure 1:**
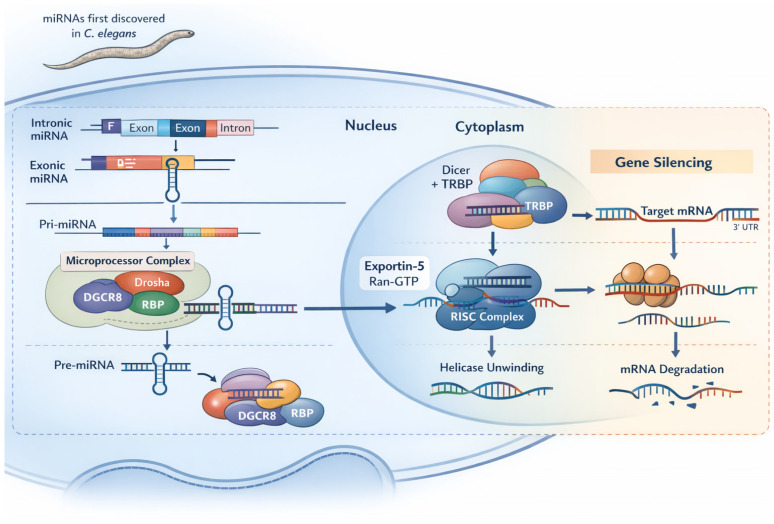
Canonical microRNA (miRNA) biogenesis and mechanism of action. miRNA genes can be located within introns or exons of protein-coding genes or organized in polycistronic clusters. Most miRNAs are transcribed by RNA polymerase II as primary miRNA transcripts (pri-miRNAs), which form hairpin structures in the nucleus. Pri-miRNAs are processed by the microprocessor complex, composed of Drosha and its cofactor DGCR8 (with associated RNA-binding proteins), to generate precursor miRNAs (pre-miRNAs). Pre-miRNAs are exported to the cytoplasm by Exportin-5 in a Ran-GTP-dependent manner. In the cytoplasm, pre-miRNAs are further cleaved by the Dicer–TRBP complex to produce an ~22-nucleotide miRNA duplex. The duplex is loaded onto Argonaute (AGO) proteins to form the RNA-induced silencing complex (RISC), followed by strand unwinding and retention of the guide miRNA. The mature miRNA directs RISC to partially complementary sequences, typically within the 3′ untranslated region (UTR) of target mRNAs, leading to translational repression and/or mRNA destabilization and degradation.

**Figure 2:**
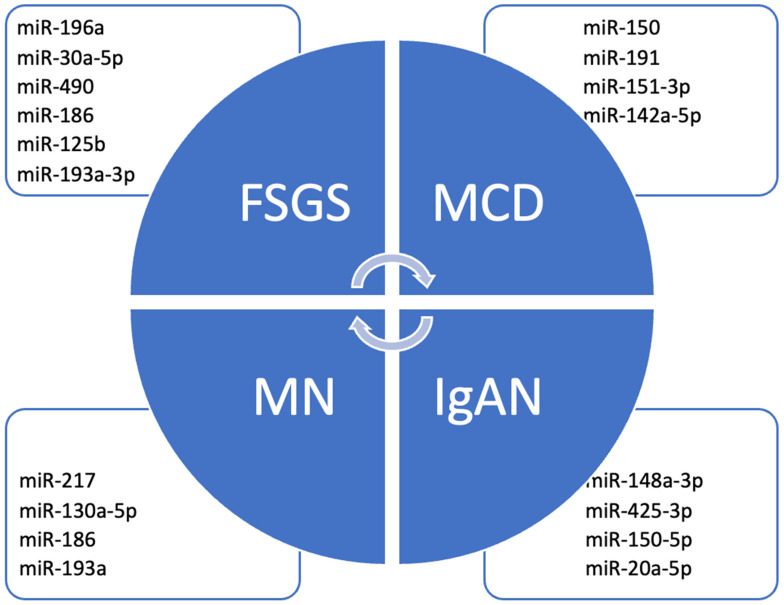
Most studied mi-RNAs in primary glomerulopathies.

However, in most cases it remains unelucidated whether these molecules are the main pathogenic determinant or if their level variations are actually a secondary response in disease specific pathways. Therefore, the aim of this review is to gather the latest information on miRNA pathophysiological implications in glomerulopathies, especially on those most studied at the moment, and the potential of existing directed therapies (see Figure 1 and Figure 2).

## 2. Methods

This review was conducted as a narrative synthesis of the available literature on microRNAs (miRNAs) in glomerular diseases, with an emphasis on primary glomerulopathies and selected secondary conditions of high clinical relevance.

This review was conducted as a narrative review and therefore no PRISMA flow diagram was applied.

A comprehensive literature search was performed using PubMed/MEDLINE, Scopus, and Web of Science databases. The search covered articles published between January 2005 and October 2025.

Search terms included combinations of keywords and MeSH terms such as: “microRNA” OR “miRNA” AND “glomerulopathy” OR “glomerulonephritis” OR “focal segmental glomerulosclerosis” OR “minimal change disease” OR “membranous nephropathy” OR “IgA nephropathy” OR “lupus nephritis” OR “diabetic nephropathy”. Afterwards, based on the initial search results, we conducted a secondary literature search, with combinations of specific micro-RNAs (e.g., “miR-150, miR-193”) and specific glomerular diseases (e.g., “focal segmental glomerulosclerosis, lupus nephritis”).

All references found by one of the authors had to be approved by another or even a third one.

Inclusion criteria comprised original experimental studies, human observational or interventional studies, reviews and translational research. Exclusion criteria included non-English articles and studies without experimental or clinical validation.

## 3. miRNAs in Glomerulopathies

Over the past decade, advances in molecular biology have clarified the central role of miRNAs in glomerular biology and disease. miRNAs are essential for nephrogenesis and the maintenance of glomerular homeostasis; deletion of the miRNA-processing enzymes Dicer or Drosha in podocytes results in severe proteinuria and glomerulosclerosis. Specific miRNA clusters, such as miR-30, regulate podocyte apoptosis and cytoskeletal integrity, while other miRNAs support glomerular endothelial and mesangial cell function by modulating endothelial–mesenchymal transition, mesangial hypertrophy, immune responses, fibrosis, and extracellular matrix turnover. Dysregulation of these pathways contributes to the onset and progression of glomerulopathies, and tissue-specific miRNA expression patterns provide insights into disease classification and prognosis.

In current clinical practice, biomarkers used in glomerular diseases are largely limited to autoantibodies and serum complement components, which have variable diagnostic and prognostic value, kidney biopsy therefore remaining the diagnostic gold standard. miRNAs are attractive biomarker candidates because they are stable, readily detectable by accessible techniques such as quantitative PCR, and measurable in blood, urine, and tissue biopsy samples. Although numerous miRNAs have been proposed as biomarkers across glomerular diseases, only a limited number have been independently validated, and results to date have been inconsistent, limiting their current translation into routine clinical practice. Accordingly, this section is organized by most common glomerular diseases, summarizing miRNAs implicated in disease pathogenesis.

### 3.1. miRNA Candidates in Focal Segmental Glomerulosclerosis (FSGS)

Focal segmental glomerulosclerosis (FSGS) is a leading cause of nephrotic syndrome in both adults and children and is characterized by focal and segmental glomerular scarring [7]. Growing evidence indicates that miRNAs play critical roles in disease pathogenesis, progression, and clinical stratification, serving as non-invasive biomarkers and potential therapeutic targets. Collectively, these studies underscore the translational potential of miRNAs as non-invasive diagnostic and prognostic markers (see Table 1).

#### 3.1.1. Plasma (Circulating) miRNAs

Several circulating miRNAs have demonstrated diagnostic and prognostic relevance in FSGS. Panels including miR-186, miR-125b, and miR-193a-3p differentiate FSGS from other glomerular diseases with good sensitivity and specificity, while exosomal miR-186-5p may act as both a biomarker and a circulating pathogenic mediator [11,12]. Plasma levels of miR-17, miR-19b, and miR-106a are significantly reduced in patients with combined FSGS variants compared with not otherwise specified or perihilar subtypes, linking miRNA profiles to histological classification. In contrast, plasma miR-342 correlates positively with proteinuria and inversely with renal function, supporting its role as a prognostic biomarker. In addition, plasma and serum miR-192 and miR-205 are elevated in FSGS relative to minimal change disease and healthy controls and correlate with proteinuria and interstitial fibrosis, consistent with their involvement in pro-fibrotic signaling [13].

#### 3.1.2. Urinary miRNAs

Urinary miRNAs represent robust non-invasive indicators of disease activity and progression. Urinary miR-196a, miR-30a-5p, and miR-490 distinguish active FSGS from remission and reflect corticosteroid responsiveness [14]. Urinary exosomal miRNAs—including miR-21, miR-30a, miR-193a, miR-196a, and miR-200a—differentiate FSGS from diabetic kidney disease [15]. Recent studies further show that urinary miR-1915 and miR-663 are significantly downregulated in FSGS compared with minimal change disease, whereas urinary miR-155 is upregulated and reflects renal inflammation and fibrosis. Notably, urinary miR-1915 correlates negatively with proteinuria, while miR-663 correlates positively with proteinuria and negatively with estimated glomerular filtration rate (eGFR). Urinary exosomal miR-193a is markedly elevated in FSGS and predicts more rapid disease progression, consistent with its role in podocyte dedifferentiation via repression of WT1 [13].

#### 3.1.3. Histologic (Tissue) miRNAs

Histologic and spatial transcriptomic analyses have identified tissue-specific miRNA signatures associated with FSGS severity. miR-21-5p, miR-146b-5p, and miR-192-5p are markedly upregulated in injured glomeruli and correlate with disease progression [16]. These miRNAs promote pro-fibrotic and pro-inflammatory pathways, including TGF-β/Smad signaling. Additional tissue-enriched miRNAs, such as miR-150 and miR-155, drive podocyte inflammation, oxidative stress, and fibrogenesis, whereas downregulation of protective miRNAs, particularly the miR-30 family and miR-106a, compromises podocyte integrity and survival. Emerging candidates, including miR-1470 and miR-4483, have also been implicated in extracellular matrix remodeling and renal fibrosis.

The robustness of these findings varies. While patient biopsy and fluid-based studies provide high-level evidence for biomarker discovery [12,13,14,16,17], mechanistic insights are largely supported by animal models and in vitro assays [18,19,20,21,22,23,24].

Nevertheless, the convergence of these translational data with recent clinical studies strongly supports the biological and diagnostic relevance of specific urinary, plasma, and exosomal miRNAs—including miR-1915, miR-663, miR-155, miR-193a, miR-342, and miR-192—in the pathogenesis and progression of FSGS [25,26,27,28,29,30].

### 3.2. miR in Minimal Change Disease (MCD)

Recent studies highlight a central role for miRNAs in minimal change disease (MCD) and pediatric nephrotic syndrome, with implications for diagnosis, disease monitoring, therapeutic response, and pathogenesis.

#### 3.2.1. Plasma (Circulating) miRNAs

Circulating miRNA panels, including miR-150, miR-191, and miR-151-3p, effectively distinguish MCD from other nephropathies and correlate with proteinuria and steroid responsiveness [10,11,12,13,14,15,16,17,18,19,20,21,22,23,24,25,26,27,28,29,30,31,32,33,34,35,36,37,38]. In pediatric nephrotic syndrome, panels comprising miR-142a-5p, miR-181a-5p, and miR-150a-5p demonstrate the highest diagnostic accuracy, supporting their use in disease stratification and early diagnosis [38].

#### 3.2.2. Urinary miRNAs

Urinary and urinary exosomal miRNAs provide robust non-invasive biomarkers in MCD. Exosomal miRNAs such as miR-194-5p, miR-23b-3p, and miR-30a-5p differentiate MCD from other glomerular diseases and correlate with disease activity and treatment response [10,11,12,13,14,15,16,17,18,19,20,21,22,23,24,25,26,27,28,29,30,31,32,33,34,35,36,37,38]. In particular, urinary miR-30a-5p and miR-151-3p are associated with steroid sensitivity and treatment outcomes, underscoring their value for monitoring therapeutic response.

#### 3.2.3. Histologic (Tissue) miRNAs

At the tissue level, miRNAs exert both protective and pathogenic effects on podocytes. Protective miRNAs, including miR-499 and miR-204-5p, preserve podocyte structure by targeting calcineurin and cathepsin D, respectively, thereby maintaining foot process integrity and nephrin stability [8,39]. In contrast, pathogenic miRNAs such as miR-17/miR-17-5p, miR-155, and miR-433 promote podocyte injury, disease progression, and fibrotic responses. Dysregulation of miR-200 and miR-205 correlates with steroid resistance, identifying these miRNAs as potential predictors of treatment failure [9,40].

In addition, immune-modulating miRNAs, including miR-24 and miR-27, contribute to Th2 polarization and IL-4/IL-13 overproduction, central mechanisms in the immunopathogenesis of nephrotic syndrome. Together, these tissue-level miRNA alterations link immune dysregulation with podocyte injury in MCD [41,42,43].

Collectively, these findings underscore the multifaceted roles of miRNAs in MCD, from diagnosis and monitoring to therapeutic intervention, supporting their integration into personalized medicine strategies for pediatric nephrotic syndrome (see Table 2) [44].

### 3.3. microRNAs in Membranous Nephropathy

Membranous nephropathy (MN) is a leading cause of primary nephrotic syndrome in adults and is increasingly recognized as a disease driven in part by miRNA dysregulation, affecting podocyte survival, immune signaling, and progression of renal injury.

#### 3.3.1. Plasma (Circulating) miRNAs

Circulating miRNAs with protective regulatory roles are reduced in MN. Plasma levels of miR-106a, miR-19b, and miR-17 are decreased in idiopathic MN and correlate with increased PTEN expression, supporting their involvement in pathways that preserve podocyte homeostasis [45]. These circulating miRNAs therefore reflect loss of protective signaling and may serve as biomarkers of disease activity.

#### 3.3.2. Urinary miRNAs

Urinary miRNAs, particularly exosomal and podocyte-derived microparticle miRNAs, represent promising non-invasive biomarkers in MN. A panel of urinary exosomal miRNAs—including miR-155-5p, miR-23b-5p, and miR-509-3p—was differentially expressed in MN patients compared with controls, demonstrating diagnostic potential [46]. In addition, miR-186 was found to be altered in urinary podocyte-derived microparticles, further supporting the utility of urinary miRNA profiling for disease detection and monitoring [47].

#### 3.3.3. Histologic (Tissue) miRNAs

At the tissue level, miRNAs exert both protective and pathogenic effects on podocytes and glomerular structure. Protective miRNAs such as miR-217, miR-130a-5p, and miR-186 attenuate podocyte apoptosis by targeting pro-apoptotic pathways involving TNFSF11, PLA2R, and TLR4, respectively; their downregulation in MN reflects loss of intrinsic protective mechanisms [48,49,50].

In contrast, miR-193a is consistently upregulated in MN and suppresses key podocyte-stabilizing genes, including WT1, PODXL, and NPHS1, leading to podocyte injury, proteinuria, and poor renal outcomes. Elevated miR-193a levels correlate with proteinuria and renal dysfunction, establishing it as a strong prognostic marker [51,52].

Recent studies have identified a novel pathogenic axis involving miR-192-5p and miR-378a-3p in idiopathic MN. miR-192-5p is upregulated in glomeruli and urine and is released by glomerular endothelial cells via exosomes that target podocytes, suppressing nephronectin (NPNT) expression in the glomerular basement membrane. This results in GBM structural alterations, increased permeability, and proteinuria. Experimental loss of NPNT recapitulates key MN features, implicating endothelial- and podocyte-derived miRNAs as early drivers of disease pathogenesis [53,54,55].

Non-coding RNA networks in MN.

Beyond miRNAs, long non-coding RNAs (lncRNAs) and circular RNAs (circRNAs) modulate miRNA activity and contribute to podocyte apoptosis in MN. circ_0000524 and circ_CDYL promote podocyte injury by suppressing miR-500a-5p and miR-149-5p, respectively, thereby enhancing CXCL16- and TNFSF11-mediated apoptosis [56,57]. Similarly, MN-associated lncRNAs regulate podocyte survival through miRNA-dependent pathways, including the miR-217–TLR4 axis [58],. These ceRNA networks act as upstream regulators of miRNA signaling and represent emerging diagnostic and therapeutic targets [59]. Their dual role as regulators and biomarkers underscores the need for further mechanistic studies to validate their translational potential in clinical nephrology (see Table 3) [59].

### 3.4. IgA Nephropathy

IgA nephropathy (IgAN) is characterized by mesangial IgA deposition leading to immune-mediated glomerular injury, hematuria, proteinuria, and progressive renal dysfunction. Advances in molecular profiling have identified miRNAs as key regulators of IgAN pathogenesis, with diagnostic and prognostic relevance [60].

#### 3.4.1. Plasma (Circulating) miRNAs

Several circulating miRNAs are dysregulated in IgAN and correlate with disease severity. Plasma miR-148a-3p and miR-425-3p are significantly elevated in biopsy-proven IgAN and correlate negatively with glomerular filtration rate (GFR), suggesting an association with disease progression [61]. Additional studies identified elevated plasma miR-148a-3p, miR-150-5p, miR-20a-5p, and miR-425-3p in early IgAN compared with advanced disease, supporting their utility as early disease biomarkers markers [62].

Serum let-7b levels are also increased in IgAN and, alone or in combination with miR-148b, predict faster renal function decline and progression to end-stage kidney disease despite immunosuppressive therapy [63]. In contrast, reduced serum miR-192 levels are associated with more severe fibrosis, tubular atrophy, and inflammation, whereas higher levels correlate with slower disease progression [64]. Decreased miR-33a-5p levels in serum further parallel worsening proteinuria and declining renal function [65].

#### 3.4.2. Urinary miRNAs

Urinary miRNAs provide robust non-invasive biomarkers for IgAN diagnosis and risk stratification. Profiling of urinary sediment identified multiple dysregulated miRNAs, with miR-16, miR-26a, and miR-150 being upregulated and miR-204, miR-431, and miR-555 downregulated in IgAN [66]. Urinary miR-221 correlates with estimated GFR, while miR-204 demonstrates high diagnostic accuracy. Consistently, urinary exosomal miR-204 is markedly reduced in IgAN, particularly in patients at high risk of progression [67].

Further validation studies identified urinary miR-106a as a highly sensitive diagnostic marker for IgAN [68]. Urinary miR-21 levels are also elevated and correlate with histological injury and future renal function decline, reinforcing their value for disease monitoring. Reduced urinary miR-33a-5p mirrors disease severity and progression [69].

#### 3.4.3. Histologic (Tissue) miRNAs

At the tissue level, miRNAs contribute directly to immune dysregulation, aberrant IgA1 glycosylation, and renal remodeling. miR-148b is upregulated in peripheral blood mononuclear cells of IgAN patients and, together with miR-374b and let-7b, regulates key glycosyltransferases (C1GALT1 and GALNT2), promoting production of galactose-deficient IgA1 [70].

Intrarenal miR-21 expression is consistently increased in IgAN and correlates with histological damage and progressive renal decline, highlighting its role as a pathogenic and prognostic marker [71]. miR-590-3p targets HMGB2 and is associated with disease severity and serum gd-IgA1 levels [72]. miR-214-3p promotes mesangial proliferation and renal injury via PTEN/JNK signaling, while its inhibition attenuates disease in experimental models [73].

Additionally, miR-150-5p is upregulated in tonsillar tissue of IgAN patients with hematuria and correlates with hypertension, dyslipidemia, inflammatory markers, and reduced eGFR, linking mucosal immune activation with renal injury [74]. Reduced miR-33a-5p expression across renal tissue parallels proteinuria and disease severity [74].

### 3.5. miRNA in Lupus Nephritis

Lupus nephritis (LN) is a severe manifestation of systemic lupus erythematosus (SLE) and a major determinant of morbidity and progression to kidney failure. Although renal biopsy remains the diagnostic gold standard, its invasiveness and the limited sensitivity of conventional laboratory markers have driven the search for reliable, minimally invasive biomarkers. In this context, miRNAs have emerged as key regulators of immune dysregulation and renal injury in LN [75].

#### 3.5.1. Plasmatic (Circulating) miRNAs

LN is characterized by a distinct circulating miRNA profile reflecting systemic autoimmunity. miR-146a is consistently downregulated, resulting in hyperactivation of NF-κB and type I interferon signaling, while miR-155 is upregulated and promotes pro-inflammatory cytokine production and T-cell activation [75].

A recent systematic review and meta-analysis including over 1000 patients demonstrated strong diagnostic performance of circulating miRNAs for LN, with pooled sensitivity of 0.85, specificity of 0.83, and an AUC of 0.91 [76]. Among these, miR-181a, miR-223, and miR-146a showed superior diagnostic accuracy, particularly when combined into multi-miRNA panels, supporting their role as reliable noninvasive biomarkers for early LN detection [76].

Serum exosomal miRNAs have further refined diagnostic precision. hsa-miR-497-5p and hsa-miR-6515-5p were significantly elevated in LN compared with SLE patients without renal involvement. Their combined expression achieved an AUC of 0.798 overall and 0.844 in patients with only mild proteinuria, highlighting sensitivity for early disease. Both miRNAs correlated positively with inflammatory cytokines such as IFN-γ and IL-8, and bioinformatic analyses implicated them in immune and MAPK signaling pathways [77].

#### 3.5.2. Urinary miRNAs

Urinary biomarkers provide insight into renal-specific injury in LN. Studies evaluating urinary extracellular vesicles (uEVs) demonstrated significantly increased levels of podocyte-derived uEVs in patients with active LN, alongside elevated urinary cytokines including IL-6, IL-8, IFN-γ, and CCL-2 [78]. Podocyte-derived uEVs correlated with proteinuria, albuminuria, and inflammatory mediator levels [78].

While individual urinary biomarkers showed modest predictive value, integrative models combining podocyte uEVs with urinary cytokine profiles achieved excellent discrimination of active LN (AUC up to 0.88, sensitivity 80%, specificity 94%), underscoring the value of multimodal urinary signatures in disease monitoring [78].

#### 3.5.3. Histologic (Tissue) miRNAs

At the tissue level, inflammatory miRNAs such as miR-146a and miR-155 are upregulated within renal compartments and immune cells, modulating NF-κB and interferon-driven pathways. Their expression correlates with histologic activity indices and clinical disease severity, reinforcing their role in LN pathogenesis and as markers of renal inflammation [75].

### 3.6. miRNAs in Diabetic Nephropathy

Diabetic nephropathy (DN) is characterized by progressive glomerulosclerosis and tubulointerstitial fibrosis driven by metabolic, inflammatory, and profibrotic signaling. miRNAs play central roles in regulating extracellular matrix (ECM) accumulation, inflammation, and fibrotic remodeling [79,80].

#### 3.6.1. Plasmatic (Circulating) miRNAs

Although most DN-associated miRNAs have been studied at the tissue level, circulating miRNAs such as miR-21 and miR-192 reflect ongoing fibrotic activity and correlate with disease severity, suggesting potential utility as noninvasive biomarkers of progressive diabetic kidney disease [79,80].

#### 3.6.2. Urinary miRNAs

Urinary miRNAs associated with fibrotic signaling, including miR-21 and members of the miR-29 family, mirror intrarenal expression patterns and have been proposed as biomarkers of renal fibrosis and disease progression in DN, although further validation is required [79,80].

#### 3.6.3. Histologic (Tissue) miRNAs

Histologic studies consistently demonstrate upregulation of miR-192 and miR-21 in both glomerular and tubular compartments in DN. miR-192 is induced by TGF-β1 and promotes fibrosis by repressing ZEB2, leading to increased collagen type I and III expression. miR-21 similarly amplifies TGF-β/Smad signaling, driving ECM accumulation, inflammation, and tubulointerstitial fibrosis. miR-377 further contributes to oxidative stress and matrix expansion [79,80,81].

Conversely, the miR-29 family—particularly miR-29b—is downregulated in DN and normally functions as an antifibrotic regulator by targeting multiple collagen and ECM transcripts; its loss exacerbates fibrotic remodeling [79,80,81].

Recent work has identified a novel RAGE-dependent regulatory axis involving miR-214 and its host noncoding RNA Dnm3os. In RAGE-deficient diabetic mice, renal miR-214 expression was increased and exerted antifibrotic and anti-inflammatory effects by directly targeting DIAPH1, a key downstream mediator of RAGE signaling. In contrast, Dnm3os promoted fibrosis and inflammation in mesangial cells. Overexpression of miR-214 attenuated renal fibrosis in diabetic mouse models, while human DN kidney tissue demonstrated increased DNM3OS expression, underscoring translational relevance [82].

Together, these findings highlight a dual regulatory mechanism in DN, in which miR-214 acts as a protective modulator while Dnm3os promotes fibrotic progression, identifying the miR-214–DIAPH1 axis as a promising therapeutic target [82].

**Figure 3:**
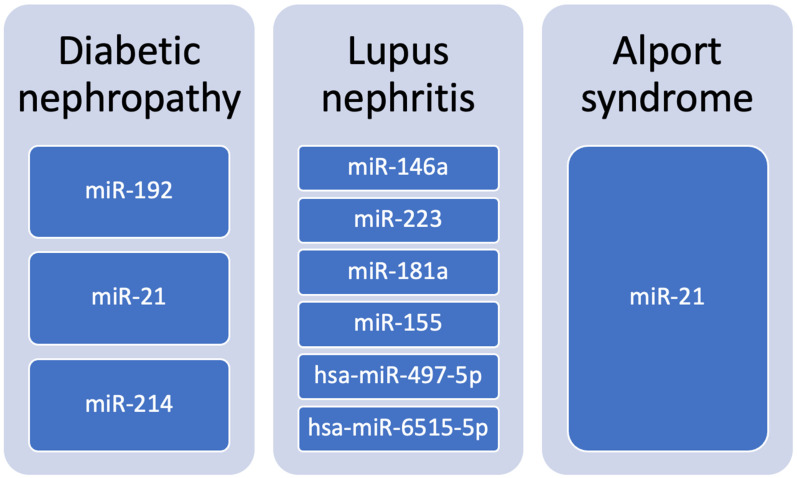
mi-RNAs involved in diabetic nephropathy, lupus nephritis and Alport syndrome.

We have summarized the involvement of different microRNAs in diabetic nephropathy, lupus nephritis and Alport syndrome in Figure 3.

## 4. RNA-Based Therapeutics Targeting miRNAs

RNA interference (RNAi) has emerged as both a powerful research tool and a promising therapeutic strategy, underscored by the FDA approval of the first siRNA-based drug, patisiran, in 2018 for the treatment of hereditary transthyretin amyloidosis. This approval represents a major milestone in the clinical translation and broader development of RNAi-based therapeutics. In kidney diseases, multiple miRNA-based approaches have demonstrated therapeutic potential by modulating key signaling pathways involved in fibrosis, inflammation, and podocyte injury [83].

Several strategies exist to inhibit or mimic miRNA function. Antagomirs suppress miRNAs by binding complementary sequences, thereby relieving repression of target mRNAs. Conversely, miRNA mimics (agomirs) are single-stranded RNAs identical to endogenous microRNAs and recapitulate their function. miRNA sponges are synthetic RNAs containing multiple miRNA-binding sites that act as competitive inhibitors. In vivo, therapeutic miRNAs and antagomirs predominantly accumulate in the liver and kidney, with the highest renal levels observed in proximal tubule epithelial cells [83].

Several antagomirs have shown efficacy in chronic kidney disease by altering the ERK–MAPK pathway, thereby disrupting growth factor secretion and fibroblast survival [84,85].Anti-miR-192 therapy reduces mesangial fibronectin and collagen accumulation, attenuating glomerular fibrosis in diabetic nephropathy models [84,86].Similarly, Dicer deletion in renal interstitial mesenchymal cells enhances PDGFR-β signaling due to reduced levels of miR-9-5p, miR-344g-3p, and miR-7074-3p, identifying these miRNAs as potential antifibrotic targets [87].

Disease-specific miRNA targeting has shown benefit across multiple glomerular disorders. In focal segmental glomerulosclerosis (FSGS), crescentic glomerulonephritis, and minimal change disease, miR-193a plays a central pathogenic role, correlating with lesion severity and proteinuria; its inhibition using corticosteroids, retinoic acid, or vitamin D agonists halted disease progression in experimental models [88,89,90]. Glucocorticoid-induced upregulation of miR-30 further attenuated disease progression in FSGS and MCD, supporting its nephroprotective role [89].

In lupus nephritis (LN), modulation of immune-related miRNAs has been particularly effective. Inhibition of miR-9-5p alleviated LN by upregulating the nephroprotective factor Foxo1 [91], while restoration of miR-127-3p suppressed IFN-I signaling through JAK1 inhibition [92]. These findings align with multiple preclinical LN studies showing that miRNA mimics or antagomiRs (e.g., miR-590-3p, miR-183, miR-146a, miR-150, miR-130b) reduce proteinuria, immune activation, and fibrosis in lupus-prone mice [93,94,95].

RNAi strategies have also demonstrated efficacy in IgA nephropathy, where glomerulus-targeted siRNA delivery against p38α and NF-κB p65 reduced inflammation and extracellular matrix deposition [96]. Cemdisiran is an investigational RNA interference therapeutic that suppresses hepatic production of complement component 5 (C5), thereby potentially reducing proteinuria in IgA nephropathy. In this phase 2, 36-week, double-blind study, treatment with cemdisiran led to a 37.4% reduction in placebo-adjusted geometric mean 24 h UPCR at week 32 and was well tolerated [97].

Alport syndrome is a hereditary nephritis caused by mutations in type IV collagen and is characterized by increased expression of miR-21 in both murine and human models. miR-21 amplifies TGF-β signaling and promotes profibrotic gene expression, thereby exacerbating progressive interstitial fibrosis [10,98]. Therapeutic inhibition of miR-21 with the antisense oligonucleotide lademirsen delayed renal failure and reduced fibrosis in preclinical models [99,100].

Although lademirsen was generally well tolerated and exhibited an acceptable safety profile in a recent phase 2 randomized controlled trial, it did not significantly slow the decline of kidney function in patients at risk of rapidly progressive disease. Although differences between human disease modifiers and more genetically uniform animal models may partly explain this lack of efficacy, post hoc analyses across genetic subgroups similarly showed no meaningful difference in eGFR decline between patients treated with lademirsen and those receiving placebo [101].

Targeting microRNAs (miRNAs) offers a promising therapeutic approach in diabetic nephropathy (DN) by inhibiting pathogenic miRNAs and restoring renoprotective ones. Small-molecule inhibitors (e.g., curcumin, resveratrol) suppress profibrotic miRNAs such as miR-21, miR-221, and miR-222 [102,103]. Anti-miRNA oligonucleotides, particularly LNA-modified anti–miR-192, reduce TGF-β-driven fibrosis and ECM accumulation in diabetic models [86]. Additional strategies include miRNA sponges, miRNA inducers (e.g., baicalin, lipoxins-A4), and miRNA mimics, all showing antifibrotic or anti-inflammatory effects in preclinical studies [104,105]. Collectively, miRNA-based therapies represent a mechanistically targeted and adaptable strategy for DN treatment [102].

Despite these encouraging findings, miRNA therapeutics face significant challenges, particularly targeted delivery and off-target effects, given that a single miRNA may regulate multiple genes across tissues [106,107]. Moreover, many studies remain preclinical or early-phase, and not all miRNAs correlate directly with disease severity. Some, such as miR-204-5p, exert protective effects by preventing nephrin degradation, with pharmacologic upregulation showing renal benefit [39].

Overall, inhibition of pathogenic miRNAs (e.g., miR-193a, miR-150, miR-21) or restoration of protective miRNAs (e.g., miR-30, miR-29a, miR-23b) consistently improves podocyte health and reduces proteinuria in experimental models [108,109,110,111,112,113,114,115,116].While RNA-based therapeutics remain in early development for glomerular diseases, their specificity, scalability, and expanding clinical success underscore their strong potential as future targeted therapies [116,117].

While discussing the findings, it is important to also address the limitations of our publication. Firstly, there is a lack of references regarding certain mentioned molecules which have not been fully studied. Secondly, several of the presented studies and trials are in their initial phases so conclusions cannot be fully affirmed with a degree of certainty as to the pathogenic role these molecules exhibit in these glomerular diseases. Finally, the same case is for current therapies which are not fully studied, and their future use is still being debated until stronger evidence emerges.

## 5. Conclusions

microRNAs have emerged as key post-transcriptional regulators in renal pathophysiology, providing new insights into the mechanisms underlying glomerular diseases and opening potential avenues for diagnosis and therapy. Their disease-specific expression patterns and involvement in inflammation, fibrosis, immune dysregulation, and podocyte injury support their relevance as both biomarkers and therapeutic targets; however, their clinical applicability remains incompletely defined.

In diabetic nephropathy, altered miRNA expression has been linked to enhanced profibrotic signaling, while other miRNAs may exert antifibrotic effects, suggesting potential therapeutic strategies that remain insufficiently validated due to model heterogeneity and limited clinical evidence. Across other glomerulopathies, disease-specific miRNA patterns show both pathogenic and protective associations, although causal mechanisms are often unclear. In focal segmental glomerulosclerosis and minimal change disease, certain miRNAs are associated with podocyte injury, inflammation, fibrosis, and treatment resistance, whereas others appear to support podocyte stability, but their therapeutic modulation in vivo remains largely unproven.

In membranous nephropathy, dysregulated miRNAs have been implicated in podocyte apoptosis and basement membrane injury, while additional candidates demonstrate protective effects in experimental settings, though clinical reproducibility is limited and regulatory RNA networks remain incompletely characterized. In IgA nephropathy and lupus nephritis, miRNA signatures correlate with immune dysregulation, fibrosis, and disease activity; however, variability among patient cohorts and detection methodologies, together with a lack of longitudinal data, continues to hinder their translation into clinical practice.

Overall, while accumulating data strongly support multiple roles for miRNAs in glomerular-specific and shared downstream pathogenic pathways, evidence remains insufficient for their routine use in clinical practice, particularly as biomarkers. Many proposed miRNA biomarkers require independent replication across diverse patient populations and must demonstrate added value beyond established clinical markers such as proteinuria. In contrast, the therapeutic landscape is more encouraging, with RNA interference–based treatments beginning to enter clinical development. These approaches may be applicable across several glomerular diseases by targeting common inflammatory and fibrotic pathways. Nonetheless, the pleiotropic nature of miRNAs and their complex regulatory networks pose challenges for therapeutic design, underscoring the need for precise, cell-specific delivery strategies. Continued large-scale, multicenter studies will be essential to determine whether miRNA-based diagnostics and therapeutics can progress from experimental promise to clinically actionable tools in precision nephrology.

As of now, micro-RNAs represent a strong future direction in glomerular diseases. Several molecules show great promise, yet current therapies require further studies especially on side effects to be fully implemented, while others are yet to be studies regarding their full role and therapeutic target potential.

## Figures and Tables

**Table 1:** Summary of microRNAs implicated in Focal Segmental Glomerulosclerosis (FSGS): functional roles, samples, findings, and evidence strength.

Function	microRNA (Reference)	Sample/Model	Findings	Functional Role/Implication	Evidence Strength
Protective (therapeutic potential)	miR-499 [8]	Podocyte injury model (PAN-induced)	Overexpression alleviated podocyte injury, reversed foot process effacement, reduced calcineurin subunits (CnAα, CnAβ)	Protects podocytes; potential therapy for MCD	Strong mechanistic evidence
Conflicting biomarker data	miR-17 [9]	Pediatric NS renal tissue, serum	Reported both upregulation and downregulation in NS; may promote podocyte injury	Dysregulated expression; potential role in MCD and FSGS transition	Moderate, conflicting evidence
Pathogenic/Biomarker	miR-155 [9]	Glomeruli of active MCD patients	Significantly upregulated; aligns with prior findings	Pro-inflammatory role; possible biomarker of disease activity	Strong association
Biomarker (differential diagnosis)	miR-150 [10]	Renal tissue of pediatric patients	Significantly lower in MCD compared to other nephropathies (MPGN, RPGN, MN)	Biomarker distinguishing MCD from other GN subtypes	Strong evidence

Abbreviations: NS—nephrotic syndrome; MCD—minimal change disease; MPGN—membranoproliferative glomerulonephritis; RPGN—rapid progressive glomerulonephritis; MN—membranous nephropathy.

**Table 2:** Functional classification of microRNAs in Minimal Change Disease (MCD) and Pediatric Nephrotic Syndrome.

Function	microRNA (Reference)	Sample/Model	Findings	Functional Role/Implication	Evidence Strength
Biomarker (urinary exosomal)	miR-194-5p, miR-146b-5p, miR-378a-3p, miR-23b-3p, miR-30a-5p [36]	Urine exosomes, pediatric NS	All five upregulated in NS; levels decreased in remission; miR-194-5p and miR-23b-3p correlated with proteinuria	Biomarkers of disease activity and proteinuria severity	Strong evidence
Immune regulation	miR-24, miR-27 [41]	Pediatric INS, active phase	Downregulated in non-atopic INS; associated with Th2 dominance, ↑ IL-4, ↑ IL-13, ↑ IgE	Regulate Th2 polarization; implicated in immune dysregulation	Strong mechanistic link
Biomarkers (SSNS vs. SRNS)	miR-1, miR-215-5p, miR-335-5p, let-7a-5p [37]	Urinary samples (100 SSNS, 100 SRNS, 50 controls)	All four upregulated in NS vs. controls; SRNS showed ↓ miR-1, miR-215-5p, miR-335-5p; ↑ let-7a-5p vs. SSNS	Biomarkers to distinguish SSNS vs. SRNS	Strong clinical evidence
Diagnostic/Prognostic panel	miR-142a-5p, miR-191a-5p, miR-181a-5p, miR-30a-5p, miR-150a-5p [38]	Pediatric NS renal tissue	Panel identified new cases of NS; miR-142a-5p most reliable for diagnosis and steroid resistance	Diagnostic and prognostic panel for NS	Strong evidence
Biomarker (treatment response)	miR-30a-5p [42]	Pediatric SSNS	Upregulated in active disease; decreased after 4 weeks of therapy but remained higher vs. controls	Biomarker of disease activity and therapy response	Moderate evidence
Biomarker (steroid sensitivity)	miR-151-3p [43]	Pediatric NS, untreated vs. GC-treated	Elevated in untreated NS; reduced after glucocorticoid therapy in SSNS	Biomarker of disease progression and therapy response	Strong mechanistic evidence
Protective (podocyte stability)	miR-204-5p [39]	Experimental nephrotoxic serum model	Prevented nephrin degradation via targeting cathepsin D	Protects podocyte structural integrity	Strong mechanistic study
Pathogenic/Biomarker	miR-433, miR-200, miR-205 [40]	Pediatric SRNS vs. SSNS	miR-433 and TGFB2 upregulated in SRNS; miR-200/205 downregulated in SRNS vs. SSNS	miR-205 potential predictor of steroid responsiveness; miR-433 pathogenic	Strong clinical + mechanistic evidence

Abbreviations: NS—nephrotic syndrome; INS—idiopathic nephrotic syndrome; SSNS—steroid-sensitive nephrotic syndrome; SRNS—steroid-resistant nephrotic syndrome; TGF—tissue growth factor; GC—glucocorticoid, ↓—decrease, ↑—increase.

**Table 3:** Functional classification of microRNAs in Membranous Nephropathy (MN).

Function	microRNA (Reference)	Sample/Model	Findings	Functional Role/Implication	Evidence Strength
Protective (anti-apoptotic)	miR-217 [48]	MN renal tissue, podocytes	Downregulated in MN; targets TNFSF11 to prevent podocyte apoptosis	Protects against podocyte apoptosis; potential biomarker	Strong experimental evidence
Pathogenic (pro-apoptotic, prognostic)	miR-193a [51,52]	MN renal tissue, podocytes	Upregulated in MN; reduces WT1, PODXL, NPHS1 expression; correlates with proteinuria, ↓ GFR, poor survival	Promotes podocyte injury; prognostic marker	Strong clinical + mechanistic evidence
Protective (anti-apoptotic)	miR-130a-5p [48]	Podocyte model (Ang II stimulation)	Downregulated; regulates PLA2R to mitigate podocyte apoptosis	Protective against Ang II–induced podocyte injury	Strong mechanistic study
Protective	miR-186 [50]	MN renal tissue, podocytes	Downregulated by Ang II; negatively regulated by TLR-4; restoration protects podocytes	Protective regulator of podocyte apoptosis	Strong mechanistic evidence
Protective (circulating biomarker)	miR-106a, miR-19b, miR-17 [45]	Serum of MN patients	Significantly reduced; negatively correlated with PTEN; associated with ↑ proteinuria, creatinine, ↓ eGFR	Circulating biomarkers of MN; protect against pro-apoptotic signaling	Moderate clinical evidence
Biomarker (urinary exosomal)	Panel of 10 miRNAs (miR-664a-5p, miR-378d, miR-23b-5p, miR-155-5p, miR-497-5p, miR-509-3p, miR-30c-1-3p, miR-877-5p, miR-532-3p, miR-331-5p) [46]	Urinary exosomes from MN patients	Differential expression compared to controls	Potential diagnostic/prognostic urinary biomarkers	Moderate evidence
Biomarker (urinary MPs)	miR-186 [47]	Podocyte-derived urinary microparticles	Altered expression in MN patients vs. controls	Biomarker of podocyte injury in MN	Moderate study
Therapeutic modulation	miR-223 [54]	Animal model of MN	Upregulated by cyclophosphamide; alleviated inflammation	Mechanistic insight into immunosuppressive therapy	Experimental evidence

Abbreviations: MN—membranous nephropathy; TNFSF—tumor necrosis factor superfamily; WT—Wilms tumor; PODXL—podocalyxin-like protein, NPHS1—nephrotic syndrome type 1; GFR—glomerular filtration rate; PLA2R—phospholipase A2 receptor; TLR—toll-like receptor; PTEN—phosphatase and tensin homolog, ↓—decrease, ↑—increase.

## Data Availability

No new data were created or analyzed in this study.

## Abbreviations

The following abbreviations are used in this manuscript:

AKI	Acute kidney injury
apoA1-b	apolipoprotein A1-b
CKD	Chronic kidney disease
CN	calcineurin
DN	Diabetic nephropathy
EMT	epithelial-to-mesenchymal transition
FSGS	Focal-segmental glomerulosclerosis
GC	glucocorticoid
GFR	glomerular filtration rate
IgA	Immunoglobulin A
IgAN	IgA nephropathy
INS	Idiopathic nephrotic syndrome
LN	Lupus nephritis
MCD	Minimal change disease
miR	microRNA
MMP-13	Matrix-metalloproteinase-13
MN	Membranous nephropathy
MPGN	Membranoproliferative glomerulonephritis
mRNA	Messenger RNA
NPHS1	nephrotic syndrome type 1
NS	Nephrotic syndrome
PLA2R	phospholipase A2 receptor
PODXL	podocalyxin-like protein
PTEN	phosphatase and tensin homolog
RNA	Ribonucleic acid
RPGN	Rapid-progressive glomerulonephritis
SRNS	Steroid-resistant nephrotic syndrome
SSNS	Steroid-sensitive nephrotic syndrome
TGF	Tissue growth factor
TIMP1	Tissue inhibitor of metalloproteinase-1
TLR	toll-like receptor
TNFSF	tumor necrosis factor superfamily
WT	Wilms tumor

## References

[1] Miyoshi J., Zhu Z., Luo A., Toden S., Zhou X., Izumi D., Kanda M., Takayama T., Parker I.M., Wang M. (2022). A microRNA-based liquid biopsy signature for the early detection of esophageal squamous cell carcinoma: A retrospective, prospective and multicenter study. Mol. Cancer.

[2] Pérez-Moreno P., Muñoz J.P., Retamal M.A. (2025). Molecular Interplay Between Non-Coding RNAs and Connexins and Its Possible Role in Cancer. Int. J. Mol. Sci..

[3] Doser R.L., Amberg G.C., Hoerndli F.J. (2020). Reactive Oxygen Species Modulate Activity-Dependent AMPA Receptor Transport in *C. elegans*. J. Neurosci..

[4] Zhang J., Ren X., Li B., Zhao Z., Li S., Zhai W. (2025). Fecal microbiota transplantation is a promising therapy for kidney diseases. Front. Med..

[5] Chen Q., Guan X., Zuo X., Wang J., Yin W. (2016). The role of high mobility group box 1 (HMGB1) in the pathogenesis of kidney diseases. Acta Pharm. Sin. B.

[6] Movahedpour A., Khatami S.H., Karami N., Vakili O., Naeli P., Jamali Z., Shabaninejad Z., Tazik K., Behrouj H., Ghasemi H. (2022). Exosomal noncoding RNAs in prostate cancer. Clin. Chim. Acta.

[7] Rout P., Hashmi M.F., Baradhi K.M. (2025). Focal Segmental Glomerulosclerosis [Updated 11 December 2024]. StatPearls [Internet].

[8] Zhang K., Sun W., Zhang L., Xu X., Wang J., Hong Y. (2018). miR-499 Ameliorates Podocyte Injury by Targeting Calcineurin in Minimal Change Disease. Am. J. Nephrol..

[9] Charmine P., Venkatesan V., Geminiganesan S., Nammalwar B.R., Dandapani M.C. (2025). MicroRNA Expression and Target Prediction in Children with Nephrotic Syndrome. Indian J. Nephrol..

[10] Lu M., Wang C., Yuan Y., Zhu Y., Yin Z., Xia Z., Zhang C. (2015). Differentially expressed microRNAs in kidney biopsies from various subtypes of nephrotic children. Exp. Mol. Pathol..

[11] Zhang C., Zhang W., Chen H.M., Liu C., Wu J., Shi S., Liu Z.H. (2015). Plasma microRNA-186 and proteinuria in focal segmental glomerulosclerosis. Am. J. Kidney Dis..

[12] Xu X., Qu S., Zhang C., Zhang M., Qin W., Ren G., Bao H., Li L., Zen K., Liu Z. (2023). CD8 T Cell-Derived Exosomal miR-186-5p Elicits Renal Inflammation via Activating Tubular TLR7/8 Signal Axis. Adv. Sci..

[13] Sun Y., Liu S., Ding W., Zhu C., Jiang G., Li H. (2025). Recent Advances in miRNA Biomarkers for Diagnosis and Prognosis of Focal Segmental Glomerulosclerosis. Kidney Dis..

[14] Zhang W., Zhang C., Chen H., Li L., Tu Y., Liu C., Shi S., Zen K., Liu Z. (2014). Evaluation of microRNAs miR-196a, miR-30a-5P, and miR-490 as biomarkers of disease activity among patients with FSGS. Clin. J. Am. Soc. Nephrol..

[15] Trabulus S., Zor M.S., Alagoz S., Dincer M.T., Meşe M., Yilmaz E., Tahir Turanli E., Seyahi N. (2024). Profiling of five urinary exosomal miRNAs for the differential diagnosis of patients with diabetic kidney disease and focal segmental glomerulosclerosis. PLoS ONE.

[16] Williams A.M., Jensen D.M., Pan X., Liu P., Liu J., Huls S., Regner K.R., Iczkowski K.A., Wang F., Li J. (2022). Histologically resolved small RNA maps in primary focal segmental glomerulosclerosis indicate progressive changes within glomerular and tubulointerstitial regions. Kidney Int..

[17] Ramezani A., Devaney J.M., Cohen S., Wing M.R., Scott R., Knoblach S., Singhal R., Howard L., Kopp J.B., Raj D.S. (2015). Circulating and urinary microRNA profile in focal segmental glomerulosclerosis: A pilot study. Eur. J. Clin. Investig..

[18] Wu J., Zheng C., Fan Y., Zeng C., Chen Z., Qin W., Zhang C., Zhang W., Wang X., Zhu X. (2014). Downregulation of microRNA-30 facilitates podocyte injury and is prevented by glucocorticoids. J. Am. Soc. Nephrol..

[19] Wu J., Zheng C., Wang X., Yun S., Zhao Y., Liu L., Lu Y., Ye Y., Zhu X., Zhang C. (2015). MicroRNA-30 family members regulate calcium/calcineurin signaling in podocytes. J. Clin. Investig..

[20] Xiao B., Wang L.N., Li W., Gong L., Yu T., Zuo Q.F., Zhao H.W., Zou Q.M. (2018). Plasma microRNA panel is a novel biomarker for focal segmental glomerulosclerosis and associated with podocyte apoptosis. Cell Death Dis..

[21] Lu S., Dong L., Jing X., Cheng G.-Y., Zhao Z.-Z. (2020). Abnormal lncRNA CCAT1/microRNA-155/SIRT1 axis promoted inflammatory response and apoptosis of tubular epithelial cells in LPS caused acute kidney injury. Mitochondrion.

[22] Qi H., Fu J., Luan J., Jiao C., Cui X., Cao X., Zhang Y., Wang Y., Kopp J.B., Pi J. (2020). miR-150 inhibitor ameliorates adriamycin-induced focal segmental glomerulosclerosis. Biochem. Biophys. Res. Commun..

[23] He K., Zhou X., Zhao J., Du H., Guo J., Deng R., Wang J. (2024). Identification and Functional Mechanism Verification of Novel MicroRNAs Associated with the Fibrosis Progression in Chronic Kidney Disease. Biochem. Genet..

[24] Yildirim D., Bender O., Karagoz Z.F., Helvacioglu F., Bilgic M.A., Akcay A., Ruzgaresen N.B. (2021). Role of autophagy and evaluation the effects of microRNAs 214, 132, 34c and prorenin receptor in a rat model of focal segmental glomerulosclerosis. Life Sci..

[25] Gebeshuber C.A., Kornauth C., Dong L., Sierig R., Seibler J., Reiss M., Tauber S., Bilban M., Wang S., Kain R. (2013). Focal segmental glomerulosclerosis is induced by microRNA-193a and its downregulation of WT1. Nat. Med..

[26] Wang L., Wang J., Wang Z., Zhou J., Zhang Y. (2021). Higher Urine Exosomal miR-193a Is Associated with a Higher Probability of Primary Focal Segmental Glomerulosclerosis and an Increased Risk of Poor Prognosis Among Children with Nephrotic Syndrome. Front. Cell Dev. Biol..

[27] Kumar V., Paliwal N., Ayasolla K., Vashistha H., Jha A., Chandel N., Chowdhary S., Saleem M.A., Malhotra A., Chander P.N. (2019). Disruption of APOL1-miR193a Axis Induces Disorganization of Podocyte Actin Cytoskeleton. Sci. Rep..

[28] Kietzmann L., Guhr S.S., Meyer T.N., Ni L., Sachs M., Panzer U., Stahl R.A., Saleem M.A., Kerjaschki D., Gebeshuber C.A. (2015). MicroRNA-193a Regulates the Transdifferentiation of Human Parietal Epithelial Cells toward a Podocyte Phenotype. J. Am. Soc. Nephrol..

[29] Zamora G., Pearson-Shaver A.L. (2025). Minimal Change Disease [Updated 10 July 2023]. StatPearls [Internet].

[30] Floege J., Amann K. (2016). Primary glomerulonephritides. Lancet.

[31] Liu G., He L., Yang X., Tang L., Shi W., She J., Wei J. (2023). MicroRNA-155-5p Aggravates Adriamycin-Induced Focal Segmental Glomerulosclerosis through Targeting Nrf2. Nephron.

[32] Yin Q., Tang T.T., Lu X.Y., Ni W.J., Yin D., Zhang Y.L., Jiang W., Zhang Y., Li Z.L., Wen Y. (2024). Macrophage-derived exosomes promote telomere fragility and senescence in tubular epithelial cells by delivering miR-155. Cell Commun. Signal..

[33] Cai X., Xia Z., Zhang C., Luo Y., Gao Y., Fan Z., Liu M., Zhang Y. (2013). Serum microRNAs levels in primary focal segmental glomerulosclerosis. Pediatr. Nephrol..

[34] Tang S., Wang Y., Xie G., Li W., Chen Y., Liang J., Liu P., Song F., Zhou J. (2020). Regulation of Ptch1 by miR-342-5p and FoxO3 Induced Autophagy Involved in Renal Fibrosis. Front. Bioeng. Biotechnol..

[35] Huang Z., Zhang Y., Zhou J., Zhang Y. (2017). Urinary Exosomal miR-193a Can Be a Potential Biomarker for the Diagnosis of Primary Focal Segmental Glomerulosclerosis in Children. BioMed Res. Int..

[36] Chen T., Wang C., Yu H., Ding M., Zhang C., Lu X., Zhang C.Y., Zhang C. (2019). Increased urinary exosomal microRNAs in children with idiopathic nephrotic syndrome. EBioMedicine.

[37] Dandapani M.C., Venkatesan V., Charmine P., Geminiganesan S., Ekambaram S. (2022). Differential urinary microRNA expression analysis of miR-1, miR-215, miR-335, let-7a in childhood nephrotic syndrome. Mol. Biol. Rep..

[38] Bayomy N.R., Abo Alfottoh W.M., Ali Eldeep S.A., Ibrahim Mabrouk Mersal A.M.S., Abd El-Bary H.M.A., Abd El Gayed E.M. (2022). Mir-142-5p as an indicator of autoimmune processes in childhood idiopathic nephrotic syndrome and as a part of MicroRNAs expression panels for its diagnosis and prediction of response to steroid treatment. Mol. Immunol..

[39] Haddad G., Blaine J. (2025). miR-204-5p Protects Nephrin from Enzymatic Degradation in Cultured Mouse Podocytes Treated with Nephrotoxic Serum. Cells.

[40] Widiasta A., Sribudiani Y., Nugrahapraja H., Rachmadi D. (2025). miRNAs involved in the TGFB signaling as possible markers of steroid-resistant nephrotic syndrome in children. Gene Rep..

[41] Ni F.F., Liu G.L., Jia S.L., Chen R.R., Liu L.B., Li C.R., Yang J., Gao X.J. (2021). Function of miR-24 and miR-27 in Pediatric Patients With Idiopathic Nephrotic Syndrome. Front. Pediatr..

[42] Sreekumar A., Safwan G.M., Shetty S.J., Kumari S., Shenoy R.D., Shenoy V. (2023). Serum miRNA-30a-5p in Steroid Sensitive Idiopathic Nephrotic Syndrome in Indian Children: A Case-control Study. J. Clin. Diagn. Res..

[43] Xu C., Li Y. (2021). Effects of miR-151-3p-mediated GLCCl1 expression on biological function in children with nephrotic syndrome. Am. J. Transl. Res..

[44] Alok A., Yadav A. (2025). Membranous Nephropathy. StatPearls [Internet].

[45] Wu L., Zhang X., Luo L., Li X., Liu Y., Qin X. (2021). Altered expression of serum miR-106a, miR-19b, miR-17, and PTEN in patients with idiopathic membranous nephropathy. J. Clin. Lab. Anal..

[46] Zhang J., Zhu Y., Cai R., Jin J., He Q. (2020). Differential Expression of Urinary Exosomal Small RNAs in Idiopathic Membranous Nephropathy. BioMed Res. Int..

[47] Farzamikia N., Ardalan M.R., Zununivahed S. (2024). #2229 miR-186, a potential noninvasive diagnostic biomarker of Membranous nephropathy in urinary microparticles. Nephrol. Dial. Transplant..

[48] Li J., Liu B., Xue H., Zhou Q.Q., Peng L. (2017). miR-217 Is a Useful Diagnostic Biomarker and Regulates Human Podocyte Cells Apoptosis via Targeting TNFSF11 in Membranous Nephropathy. BioMed Res. Int..

[49] Liu D., Liu F., Wang X., Qiao Y., Pan S., Yang Y., Hu Y., Zhang Y., Tian F., Liu Z. (2018). MiR-130a-5p prevents angiotensin II-induced podocyte apoptosis by modulating M-type phospholipase A2 receptor. Cell Cycle.

[50] Sha W.G., Shen L., Zhou L., Xu D.Y., Lu G.Y. (2015). Down-regulation of miR-186 contributes to podocytes apoptosis in membranous nephropathy. Biomed. Pharmacother..

[51] Zhang W., Ren Y., Li J. (2019). Application of miR-193a/WT1/PODXL axis to estimate risk and prognosis of idiopathic membranous nephropathy. Ren. Fail..

[52] Li J., Chen Y., Shen L., Deng Y. (2019). Improvement of membranous nephropathy by inhibition of miR-193a to affect podocytosis via targeting WT1. J. Cell. Biochem..

[53] Müller-Deile J., Sopel N., Ohs A., Rose V., Gröner M., Wrede C., Hegermann J., Daniel C., Amann K., Zahner G. (2021). Glomerular Endothelial Cell-Derived microRNA-192 Regulates Nephronectin Expression in Idiopathic Membranous Glomerulonephritis. J. Am. Soc. Nephrol..

[54] Yao C., Ma Q., Shi Y., Zhang N., Pang L. (2024). Cyclophosphamide ameliorates membranous nephropathy by upregulating miR-223 expression, promoting M2 macrophage polarization and inhibiting inflammation. Technol. Health Care.

[55] Hejazian S.M., Ardalan M., Shoja M.M., Samadi N., Zununi Vahed S. (2020). Expression Levels of miR-30c and miR-186 in Adult Patients with Membranous Glomerulonephritis and Focal Segmental Glomerulosclerosis. Int. J. Nephrol. Renov. Dis..

[56] Sun Z., Xu Q., Ma Y., Yang S., Shi J. (2021). Circ_0000524/miR-500a-5p/CXCL16 axis promotes podocyte apoptosis in membranous nephropathy. Eur. J. Clin. Investig..

[57] Qiu D., Zhao N., Chen Q., Wang M. (2022). Knockdown of circ_CDYL Contributes to Inhibit Angiotensin II-Induced Podocytes Apoptosis in Membranous Nephropathy via the miR-149-5p/TNFSF11 Pathway. J. Cardiovasc. Pharmacol..

[58] Jin L.W., Pan M., Ye H.Y., Zheng Y., Chen Y., Huang W.W., Xu X.Y., Zheng S.B. (2019). Down-regulation of the long non-coding RNA XIST ameliorates podocyte apoptosis in membranous nephropathy via the miR-217-TLR4 pathway. Exp. Physiol..

[59] Wang L., Feng X. (2024). Construction of a drug, miRNA, and transcription factor regulatory network in membranous nephropathy based on oxidative stress-related genes. J. Taibah Univ. Sci..

[60] Rout P., Limaiem F., Hashmi M.F. (2025). IgA Nephropathy (Berger Disease) [Updated 22 April 2024]. StatPearls [Internet].

[61] Przybyciński J., Czerewaty M., Kwiatkowska E., Dziedziejko V., Safranow K., Domański L., Pawlik A. (2025). MicroRNAs miR-148a-3p, miR-425-3p, and miR-20a-5p in Patients with IgA Nephropathy. Genes.

[62] Wu J., Zhang H., Wang W., Zhu M., Qi L.W., Wang T., Cheng W., Zhu J., Shan X., Huang Z. (2018). Plasma microRNA signature of patients with IgA nephropathy. Gene.

[63] Kouri N.M., Stangou M., Lioulios G., Mitsoglou Z., Serino G., Chiurlia S., Cox S.N., Stropou P., Schena F.P., Papagianni A. (2021). Serum Levels of miR-148b and Let-7b at Diagnosis May Have Important Impact in the Response to Treatment and Long-Term Outcome in IgA Nephropathy. J. Clin. Med..

[64] Fan Q., Lu R., Zhu M., Yan Y., Guo X., Qian Y., Zhang L., Dai H., Ni Z., Gu L. (2019). Serum miR-192 Is Related to Tubulointerstitial Lesion and Short-Term Disease Progression in IgA Nephropathy. Nephron.

[65] Liu L., Duan A., Guo Q., Sun G., Cui W., Lu X., Yu H., Luo P. (2021). Detection of microRNA-33a-5p in serum, urine and renal tissue of patients with IgA nephropathy. Exp. Ther. Med..

[66] Szeto C.C., Wang G., Ng J.K., Kwan B.C., Mac-Moune Lai F., Chow K.M., Luk C.C., Lai K.B., Li P.K. (2019). Urinary miRNA profile for the diagnosis of IgA nephropathy. BMC Nephrol..

[67] Pawluczyk I., Nicholson M., Barbour S., Er L., Selvaskandan H., Bhachu J.S., Barratt J. (2021). A Pilot Study to Predict Risk of IgA Nephropathy Progression Based on miR-204 Expression. Kidney Int. Rep..

[68] Szeto C.C., Ng J.K., Fung W.W., Chan G.C., Luk C.C., Lai K.B., Wang G., Chow K.M., Mac-Moune Lai F. (2022). Urinary mi-106a for the diagnosis of IgA nephropathy: Liquid biopsy for kidney disease. Clin. Chim. Acta.

[69] Szeto C.C., Ng J.K., Fung W.W., Luk C.C., Wang G., Chow K.M., Lai K.B., Li P.K., Lai F.M. (2021). Kidney microRNA-21 Expression and Kidney Function in IgA Nephropathy. Kidney Med..

[70] Serino G., Sallustio F., Cox S.N., Pesce F., Schena F.P. (2012). Abnormal miR-148b expression promotes aberrant glycosylation of IgA1 in IgA nephropathy. J. Am. Soc. Nephrol..

[71] Xu B.Y., Meng S.J., Shi S.F., Liu L.J., Lv J.C., Zhu L., Zhang H. (2020). MicroRNA-21-5p participates in IgA nephropathy by driving T helper cell polarization. J. Nephrol..

[72] Zhai Y., Qi Y., Long X., Dou Y., Liu D., Cheng G., Xiao J., Liu Z., Zhao Z. (2019). Elevated hsa-miR-590-3p expression down-regulates HMGB2 expression and contributes to the severity of IgA nephropathy. J. Cell. Mol. Med..

[73] Li Y., Xia M., Peng L., Liu H., Chen G., Wang C., Yuan D., Liu Y., Liu H. (2021). Downregulation of miR-214-3p attenuates mesangial hypercellularity by targeting PTEN-mediated JNK/c-Jun signaling in IgA nephropathy. Int. J. Biol. Sci..

[74] Xu Y., He Y., Hu H., Xu R., Liao Y., Dong X., Song H., Chen X., Chen J. (2021). The increased miRNA-150-5p expression of the tonsil tissue in patients with IgA nephropathy may be related to the pathogenesis of disease. Int. Immunopharmacol..

[75] Lu J., Kwan B.C., Lai F.M., Tam L.S., Li E.K., Chow K.M., Wang G., Li P.K., Szeto C.C. (2012). Glomerular and tubulointerstitial miR-638, miR-198 and miR-146a expression in lupus nephritis. Nephrology.

[76] Alemayehu E., Gedefie A., Debash H., Worede A., Mulatie Z., Ebrahim E., Teshome M., Ebrahim H., Belete M.A. (2025). Circulating MiRNAs as diagnostic biomarkers of lupus nephritis in patients with systemic lupus erythematosus: A systematic review and meta-analysis. Sci. Rep..

[77] Chen F., Gao J., Shi B., Liu W., Gong J., Khan A., Sun Y., Yang P., Li Z. (2025). Identification of serum exosomal miRNA biomarkers in patients with lupus nephritis. Clin. Rheumatol..

[78] Alves L., Lemos A.P., Martins J., da Costa Fonseca S., Gaudio R.C., Lima H., Carvalho C., Ramos A., de Souza Lacerda G., da Fonseca P.B. (2025). Podocyte extracellular vesicles and immune mediators as urinary biomarkers in active lupus nephritis. Sci. Rep..

[79] Kato M., Zhang J., Wang M., Lanting L., Yuan H., Rossi J.J., Natarajan R. (2007). MicroRNA-192 in diabetic kidney glomeruli and its function in TGF-beta-induced collagen expression via inhibition of E-box repressors. Proc. Natl. Acad. Sci. USA.

[80] Wang B., Komers R., Carew R., Winbanks C.E., Xu B., Herman-Edelstein M., Koh P., Thomas M., Jandeleit-Dahm K., Gregorevic P. (2012). Suppression of microRNA-29 expression by TGF-β1 promotes collagen expression and renal fibrosis. J. Am. Soc. Nephrol..

[81] Wang Q., Wang Y., Minto A.W., Wang J., Shi Q., Li X., Quigg R.J. (2008). MicroRNA-377 is up-regulated and can lead to increased fibronectin production in diabetic nephropathy. FASEB J..

[82] Hagiwara S., Okabe J., Ziemann M., Drew B., Murakoshi M., Sourris K.C., McClelland A.D., Bose M., Ekinci E.I., Coughlan M.T. (2025). miR-214 and Its Primary Transcript Dnm3os Regulate Fibrosis and Inflammation Through RAGE Signaling in Diabetic Kidney Disease. Diabetes.

[83] Prins J., Biscans A., Jan van Zonneveld A., Frazier K.S., van der Veer E.P. (2026). RNA-based therapeutic opportunities for the treatment of kidney diseases. Nat. Rev. Nephrol..

[84] Jaswani P., Prakash S., Dhar A., Sharma R.K., Prasad N., Agrawal S. (2017). MicroRNAs Involvement in Renal Pathophysiology: A Bird’s Eye View. Indian J. Nephrol..

[85] Thum T., Gross C., Fiedler J., Fischer T., Kissler S., Bussen M., Galuppo P., Just S., Rottbauer W., Frantz S. (2008). MicroRNA-21 contributes to myocardial disease by stimulating MAP kinase signalling in fibroblasts. Nature.

[86] Putta S., Lanting L., Sun G., Lawson G., Kato M., Natarajan R. (2012). Inhibiting microRNA-192 ameliorates renal fibrosis in diabetic nephropathy. J. Am. Soc. Nephrol..

[87] Sakuma H., Maruyama K., Aonuma T., Kobayashi Y., Hayasaka T., Kano K., Kawaguchi S., Nakajima K.I., Kawabe J.I., Hasebe N. (2024). Inducible deletion of microRNA activity in kidney mesenchymal cells exacerbates renal fibrosis. Sci. Rep..

[88] Bharati J., Chander P.N., Singhal P.C. (2023). Parietal Epithelial Cell Behavior and Its Modulation by microRNA-193a. Biomolecules.

[89] Badal S.S., Danesh F.R. (2015). MicroRNAs and their applications in kidney diseases. Pediatr. Nephrol..

[90] Roman M., Nowicki M. (2024). Detailed Pathophysiology of Minimal Change Disease: Insights into Podocyte Dysfunction, Immune Dysregulation, and Genetic Susceptibility. Int. J. Mol. Sci..

[91] Yan H., Tong W. (2025). Prevention of the Progression of lupus Nephritis in MRL/lpr Mice by Modulating miR-9-5p/Foxo1 Axis. Physiol. Res..

[92] Wu L., Han X., Jiang X., Ding H., Qi C., Yin Z., Xiao J., Xiong L., Guo Q., Ye Z. (2021). Downregulation of Renal Hsa-miR-127-3p Contributes to the Overactivation of Type I Interferon Signaling Pathway in the Kidney of Lupus Nephritis. Front. Immunol..

[93] Luan J., Fu J., Chen C., Jiao C., Kong W., Zhang Y., Chang Q., Wang Y., Li D., Illei G.G. (2019). LNA-anti-miR-150 ameliorated kidney injury of lupus nephritis by inhibiting renal fibrosis and macrophage infiltration. Arthritis Res. Ther..

[94] Huang J., Xu X., Wang X., Yang J., Xue M., Yang Y., Zhang R., Yang X., Yang J. (2022). MicroRNA-590-3p inhibits T helper 17 cells and ameliorates inflammation in lupus mice. Immunology.

[95] Li X., Luo F., Li J., Luo C. (2019). MiR-183 delivery attenuates murine lupus nephritis-related injuries via targeting mTOR. Scand. J. Immunol..

[96] Wang Y., Wu Q., Wang J., Li L., Sun X., Zhang Z., Zhang L. (2020). Co-delivery of p38α MAPK and p65 siRNA by novel liposomal glomerulus-targeting nano carriers for effective immunoglobulin a nephropathy treatment. J. Control. Release.

[97] Barratt J., Liew A., Yeo S.C., Fernström A., Barbour S.J., Sperati C.J., Villanueva R., Wu M.J., Wang D., Borodovsky A. (2024). Cemdisiran Phase 2 Study Investigators and Collaborators. Phase 2 Trial of Cemdisiran in Adult Patients with IgA Nephropathy: A Randomized Controlled Trial. Clin. J. Am. Soc. Nephrol..

[98] Gomez I.G., MacKenna D.A., Johnson B.G., Kaimal V., Roach A.M., Ren S., Nakagawa N., Xin C., Newitt R., Pandya S. (2015). Anti-microRNA-21 oligonucleotides prevent Alport nephropathy progression by stimulating metabolic pathways. J. Clin. Investig..

[99] Li F., Dai B., Ni X. (2020). Long non-coding RNA cancer susceptibility candidate 2 (CASC2) alleviates the high glucose-induced injury of CIHP-1 cells via regulating miR-9-5p/PPARγ axis in diabetes nephropathy. Diabetol. Metab. Syndr..

[100] Rubel D., Boulanger J., Craciun F., Xu E.Y., Zhang Y., Phillips L., Callahan M., Weber W., Song W., Ngai N. (2022). Anti-microRNA-21 Therapy on Top of ACE Inhibition Delays Renal Failure in Alport Syndrome Mouse Models. Cells.

[101] Gale D.P., Gross O., Wang F., Esteban de la Rosa R.J., Hall M., Sayer J.A., Appel G., Hariri A., Liu S., Maski M. (2024). A Randomized Controlled Clinical Trial Testing Effects of Lademirsen on Kidney Function Decline in Adults with Alport Syndrome. Clin. J. Am. Soc. Nephrol..

[102] Yarahmadi A., Shahrokhi S.Z., Mostafavi-Pour Z., Azarpira N. (2021). MicroRNAs in diabetic nephropathy: From molecular mechanisms to new therapeutic targets of treatment. Biochem. Pharmacol..

[103] Kölling M., Kaucsar T., Schauerte C., Hübner A., Dettling A., Park J.K., Busch M., Wulff X., Meier M., Scherf K. (2017). Therapeutic miR-21 Silencing Ameliorates Diabetic Kidney Disease in Mice. Mol. Ther..

[104] Wang C., Sun Y., Liu W., Liu Y., Afzal S., Grover J., Chang D., Münch G., Li C.G., Lin S. (2022). Protective effect of the curcumin-baicalein combination against macrovascular changes in diabetic angiopathy. Front. Endocrinol..

[105] Wang B., Jha J.C., Hagiwara S., McClelland A.D., Jandeleit-Dahm K., Thomas M.C., Cooper M.E., Kantharidis P. (2014). Transforming growth factor-β1-mediated renal fibrosis is dependent on the regulation of transforming growth factor receptor 1 expression by let-7b. Kidney Int..

[106] Selvaskandan H., Pawluczyk I., Barratt J. (2023). Clinical application of microRNAs in glomerular diseases. Nephrol. Dial. Transplant..

[107] Setten R.L., Rossi J.J., Han S.P. (2019). The current state and future directions of RNAi-based therapeutics. Nat. Rev. Drug. Discov..

[108] Liu F., Chen J., Luo C., Meng X. (2022). Pathogenic Role of MicroRNA Dysregulation in Podocytopathies. Front. Physiol..

[109] Mishra A., Ayasolla K., Kumar V., Lan X., Vashistha H., Aslam R., Hussain A., Chowdhary S., Marashi Shoshtari S., Paliwal N. (2018). Modulation of apolipoprotein L1-microRNA-193a axis prevents podocyte dedifferentiation in high-glucose milieu. Am. J. Physiol. Renal Physiol..

[110] Zhou Z., Wan J., Hou X., Geng J., Li X., Bai X. (2017). MicroRNA-27a promotes podocyte injury via PPARγ-mediated β-catenin activation in diabetic nephropathy. Cell Death Dis..

[111] Zheng Z., Hu H., Tong Y., Hu Z., Cao S., Shan C., Lin W., Yin Y., Li Z. (2018). MiR-27b regulates podocyte survival through targeting adenosine receptor 2B in podocytes from non-human primate. Cell Death Dis..

[112] Badal S.S., Wang Y., Long J., Corcoran D.L., Chang B.H., Truong L.D., Kanwar Y.S., Overbeek P.A., Danesh F.R. (2016). miR-93 regulates Msk2-mediated chromatin remodelling in diabetic nephropathy. Nat. Commun..

[113] Zhao B., Li H., Liu J., Han P., Zhang C., Bai H., Yuan X., Wang X., Li L., Ma H. (2016). MicroRNA-23b Targets Ras GTPase-Activating Protein SH3 Domain-Binding Protein 2 to Alleviate Fibrosis and Albuminuria in Diabetic Nephropathy. J. Am. Soc. Nephrol..

[114] Liu Y., Li H., Liu J., Han P., Li X., Bai H., Zhang C., Sun X., Teng Y., Zhang Y. (2017). Variations in MicroRNA-25 Expression Influence the Severity of Diabetic Kidney Disease. J. Am. Soc. Nephrol..

[115] Ding H., Li J., Li Y., Yang M., Nie S., Zhou M., Zhou Z., Yang X., Liu Y., Hou F.F. (2021). MicroRNA-10 negatively regulates inflammation in diabetic kidney via targeting activation of the NLRP3 inflammasome. Mol. Ther..

[116] Lin C.L., Lee P.H., Hsu Y.C., Lei C.C., Ko J.Y., Chuang P.C., Huang Y.T., Wang S.Y., Wu S.L., Chen Y.S. (2014). MicroRNA-29a promotion of nephrin acetylation ameliorates hyperglycemia-induced podocyte dysfunction. J. Am. Soc. Nephrol..

[117] Zhang M.M., Bahal R., Rasmussen T.P., Manautou J.E., Zhong X.B. (2021). The growth of siRNA-based therapeutics: Updated clinical studies. Biochem. Pharmacol..

